# Genome-wide analysis of wheat DNA-binding with one finger (Dof) transcription factor genes: evolutionary characteristics and diverse abiotic stress responses

**DOI:** 10.1186/s12864-020-6691-0

**Published:** 2020-04-03

**Authors:** Yue Liu, Nannan Liu, Xiong Deng, Dongmiao Liu, Mengfei Li, Dada Cui, Yingkao Hu, Yueming Yan

**Affiliations:** 1 College of Life Science, Capital Normal University, Xisanhuan Beilu No. 105, 100048 Beijing, People’s Republic of China; 2 Hubei Collaborative Innovation Center for Grain Industry (HCICGI), Yangtze University, Jingzhou, 434025 China

**Keywords:** Wheat, Dof transcription factors, Phylogenetics, Evolution, Transcript expression, Abiotic stress

## Abstract

**Background:**

DNA binding with one finger (Dof) transcription factors play important roles in plant growth and abiotic stress responses. Although genome-wide identification and analysis of the DOF transcription factor family has been reported in other species, no relevant studies have emerged in wheat. The aim of this study was to investigate the evolutionary and functional characteristics associated with plant growth and abiotic stress responses by genome-wide analysis of the wheat Dof transcription factor gene family.

**Results:**

Using the recently released wheat genome database (IWGSC RefSeq v1.0), we identified 96 wheat Dof gene family members, which were phylogenetically clustered into five distinct subfamilies. Gene duplication analysis revealed a broad and heterogeneous distribution of *TaDofs* on the chromosome groups 1 to 7, and obvious tandem duplication genes were present on chromosomes 2 and 3.Members of the same gene subfamily had similar exon-intron structures, while members of different subfamilies had obvious differences. Functional divergence analysis indicated that type-II functional divergence played a major role in the differentiation of the *TaDof* gene family. Positive selection analysis revealed that the Dof gene family experienced different degrees of positive selection pressure during the process of evolution, and five significant positive selection sites (30A, 31 T, 33A, 102G and 104S) were identified. Additionally, nine groups of coevolving amino acid sites, which may play a key role in maintaining the structural and functional stability of Dof proteins, were identified. The results from the RNA-seq data and qRT-PCR analysis revealed that *TaDof* genes exhibited obvious expression preference or specificity in different organs and developmental stages, as well as in diverse abiotic stress responses. Most *TaDof* genes were significantly upregulated by heat, PEG and heavy metal stresses.

**Conclusions:**

The genome-wide analysis and identification of wheat DOF transcription factor family and the discovery of important amino acid sites are expected to provide new insights into the structure, evolution and function of the plant *Dof* gene family.

## Background

Transcription factors (TFs) involve in activating or inhibiting the activity of RNA polymerase to regulate the spatiotemporal expression of the target genes by recognizing specific DNA sequence elements present in the promoter region of the gene [1]. DNA binding with one finger (Dof) transcription factors are plant-specific. Dof proteins are generally 200–400 amino acids long with a highly conserved Dof domain of 50–52 amino acids, which is structured as a C2C2-type zinc finger that recognizes a cis-regulatory element with the common core sequence of 5′-AAAG-3′ [2–4]. Unlike the conserved N-terminal domain, a transcriptional regulatory domain at the C-terminal of Dof proteins varies greatly, which can react with different regulatory proteins or substances to activate or inhibit gene transcription [3].

The Dof domain is a bifunctional domain that mediates both DNA-protein and protein-protein interactions [5, 6]. The first protein-protein interaction was observed in the *Arabidopsis thaliana* Dof domain protein OBP1, which interacted with bZIP proteins associated with stress responses. OBPl specifically increased the binding of the OBF proteins to octopine synthase (‘ocs’) element sequences [7]. The Dof transcription factor prolamin-box binding factor (PBF) in maize can activate the gene expression of cereal storage protein by binding to the P-box present in the prolamin gene (zein) promoter. Meanwhile, PBF can interact with bZIP protein Opaque2 (O2) to activate gamma-zein expression and regulate the protein content of the endosperm in maize [5]. OsDof3 in rice regulates the gibberellin response through interaction with GAMYB [8]. The Dof protein SAD from barley can activate transcription of endosperm-specific genes through interaction with R2R3MYB protein [9]. AtDof3.2 from *A. thaliana* acts as a negative regulator of seed germination and interacts with a positive regulator of seed germination TCP14 [10].

Dof TFs have important functions in plant growth and development, as well as various environmental stress responses. The function of Dof genes in *A. thaliana* has been extensively studied, and several AtDof genes have been shown to function in plant growth and C/N metabolism [11, 12], shoot branching and seed coat formation [13], vascular tissue development and interfascicular cambium formation [14], photoperiodic control of flowering [15–17], morphogenesis and stomata functioning [18], and abiotic stress tolerance [17]. PBF (RPBF) Dof, an activator of seed storage protein genes in rice, participates in the regulation of endosperm-specific gene expression [19]. *OsDof12* regulates flowering in long-day condition, and is inhibited by dark treatment [20]. The PBF Dof in maize is involved in seed protein and starch biosynthesis [21, 22]. ZmDof3 plays an important role in maize endosperm development [23]. Maize Dof1 can activate the *PEPC* gene expression and enhance transcription from the promoters of pyruvate kinase and orthophosphate dikinase [24]. Several tomato Dof genes were found to participate in the control of flowering time and abiotic stress responses [25]. In wheat, PBF homologous genes *TaDof2* (*WPBF-A*), *TaDof3* (*WPBF-D*) and *TaDof6* (*WPBF-B*) were found to locate on the A, B and D genomes of common wheat (*Triticum aestivum* L.), respectively [26, 27]. Wheat PBF could trans-activate the transcription of the native alpha-gliadin promoter by binding to the intact prolamin-box [28]. *TaDof1* was found to participate in the process of nitrogen assimilation and control the expression of genes involved in nitrogen assimilation, specifically *GS* and *GOGAT* [29]. A recent report has shown that the *TaDof2*, *TaDof3* and *TaDof6* genes are involved in water-deficit response [30].

As a DNA-binding protein, the first Dof protein (ZmDof1) was identified and characterized in maize [31]. Subsequently, a large number of *Dof* genes have been found in different plant genomes. The number of *Dof* genes identified from genome-based surveys varies depending on the plant species, such as 36 in *A. thaliana* [3], 30 in rice [32], 26 in barley [33], 28 in sorghum [34], 27 in *Brachypodium distachyon* [35], 34 in tomato [36], 46 in maize [37], 36 in cucumber [38], 33 in pepper [39] and 29 in eggplants [40]*.* Common wheat, as an allohexaploid species, has a huge genome (up to 17 GB) and more than 85% repeat sequences, leading to the slow progress of wheat genome sequencing. Earlier work only identified 31 *Dof* genes in bread wheat [27]. Since 2018, the wheat genome project has made great progress, and the wheat genome data have been updated to IWGSC Annotation v1.0 by the International Wheat Genome Sequencing Consortium (IWGSC) through an improvement of the current wheat chromosome level assembly [41]. The completion of the sequencing of the wheat genome will accelerate the studies on the structure, evolution and function of the wheat *Dof* gene family.

In this study, using common wheat genome database (IWGSC RefSeq v1.0), we conducted a comprehensive genome-wide analysis on the structural characterization, molecular evolution and expression profiling of the wheat Dof gene family, which can provide new information for further understanding the evolution characteristics and function of plant *Dof* genes.

## Results

### Genome-wide identification of wheat *Dof* genes

Firstly, 36 and 30 Dof protein sequences from *A. thaliana* and rice were downloaded from the PlantTFDB v4.0 database (Table S1). These sequences were used as queries for searches in the recently released *Triticum aestivum* (common or bread wheat) genome database (IWGSC RefSeq v1.0). Then SMART and Pfam websites were used to further identify whether candidate sequences have conserved Dof domain. Ultimately, a total of 96 members of the Dof transcription factor gene family were identified from wheat. For convenience, these *TaDof* genes were assigned names from *TaDof1* to *TaDof96* as listed in Table S2.

The number of amino acids of the *TaDof* encoding proteins varied from 152 to 539 amino acids, their pI values ranged from 4.66 to 10.46 with an average of 8.05 and weakly alkaline, and their molecular weights were from 15.77 to 58.13 kDa, with an average of 33.38 kDa. Their detailed information is shown in Table S2. These results indicated that variations in the amino acid sequence length of Dofs may be associated with adaptation to different functional requirements and physical/chemical properties.

### Chromosome location and genes duplication of 96 *TaDof* genes

**Fig. 1:**
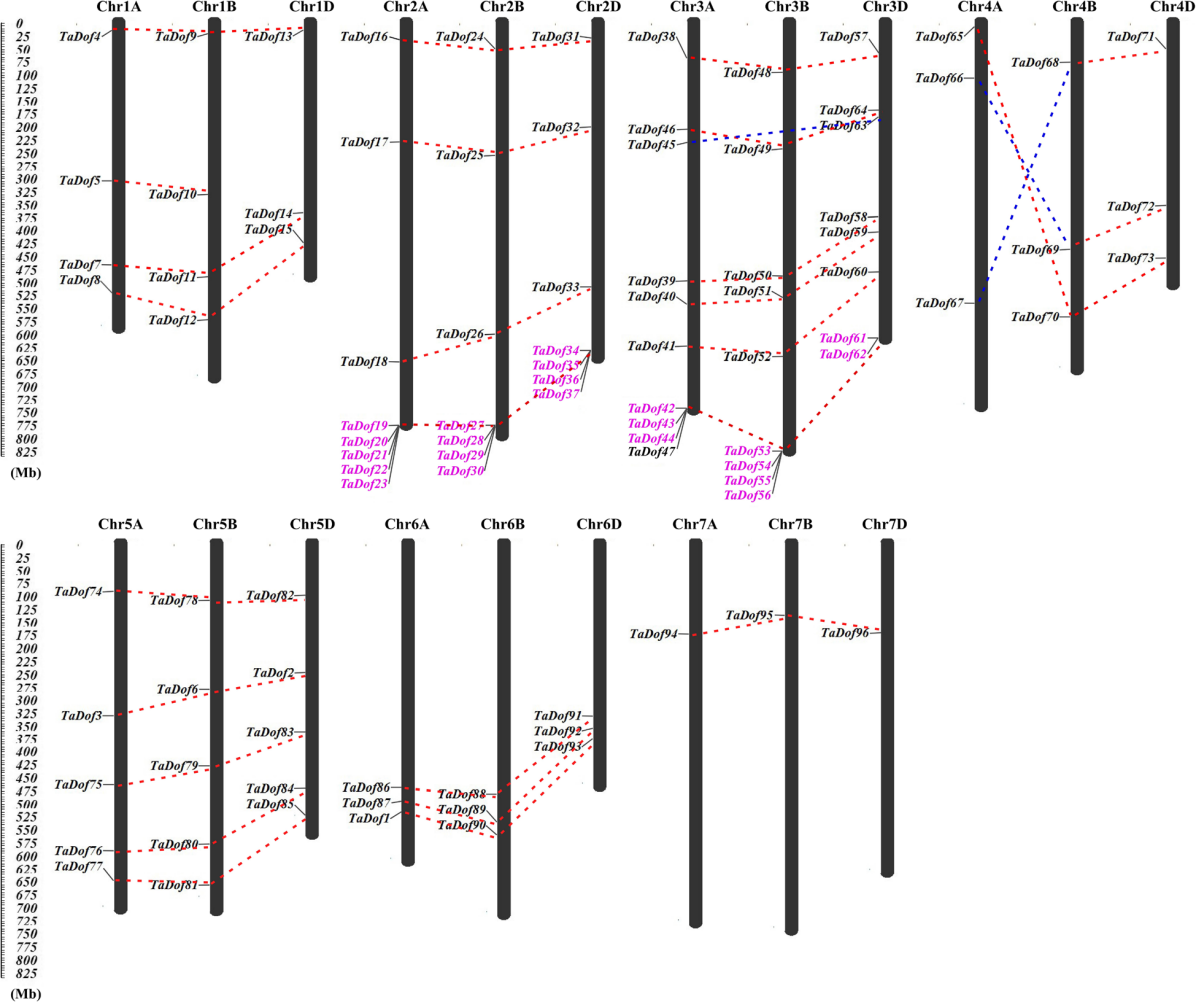
Distribution and duplication of *TaDof* genes in *Triticum aestivum* L. chromosomes. The length of chromosomes was their relative extent. Putative *TaDof* homologous genes were ligated with a red dotted line. In order to distinguish the intersecting lines, one of the red dotted lines was replaced with blue. The tandem duplicated genes were marked in pink

Based on the IWGSC database, the physical locations of the *TaDof* genes on the corresponding chromosomes are depicted in Fig. 1. All *TaDof* genes identified could be mapped on the chromosomes from 1A to 7D. Evidently, the *TaDof* genes were unevenly distributed on different chromosomes, including 34 *TaDof* genes in chromosome A, 32 in chromosome B, and 30 in chromosome D. Most *TaDofs* genes had corresponding homologous genes on the A, B, and D chromosomes. In particular, chromosome 3 with 27 *TaDof* members from *TaDof38* to *TaDof64* had the highest density, and they were closely arranged at the lower part of the chromosomes, but chromosome 7 only contained three *TaDof* genes (*TaDof94*, *TaDof95* and *TaDof96*). Interestingly, we found that the genes located on chromosome 4A were opposite to the position of the homologous genes located on chromosome 4B and chromosome 4D. In addition, segmental duplication and tandem duplication analysis revealed that *TaDofs* transcription factor family was not generated by segmental duplication, but obvious tandem duplication genes were present at the ends on the chromosomes 2 and 3.

### Subcellular localization of TaDof proteins

**Fig. 2:**
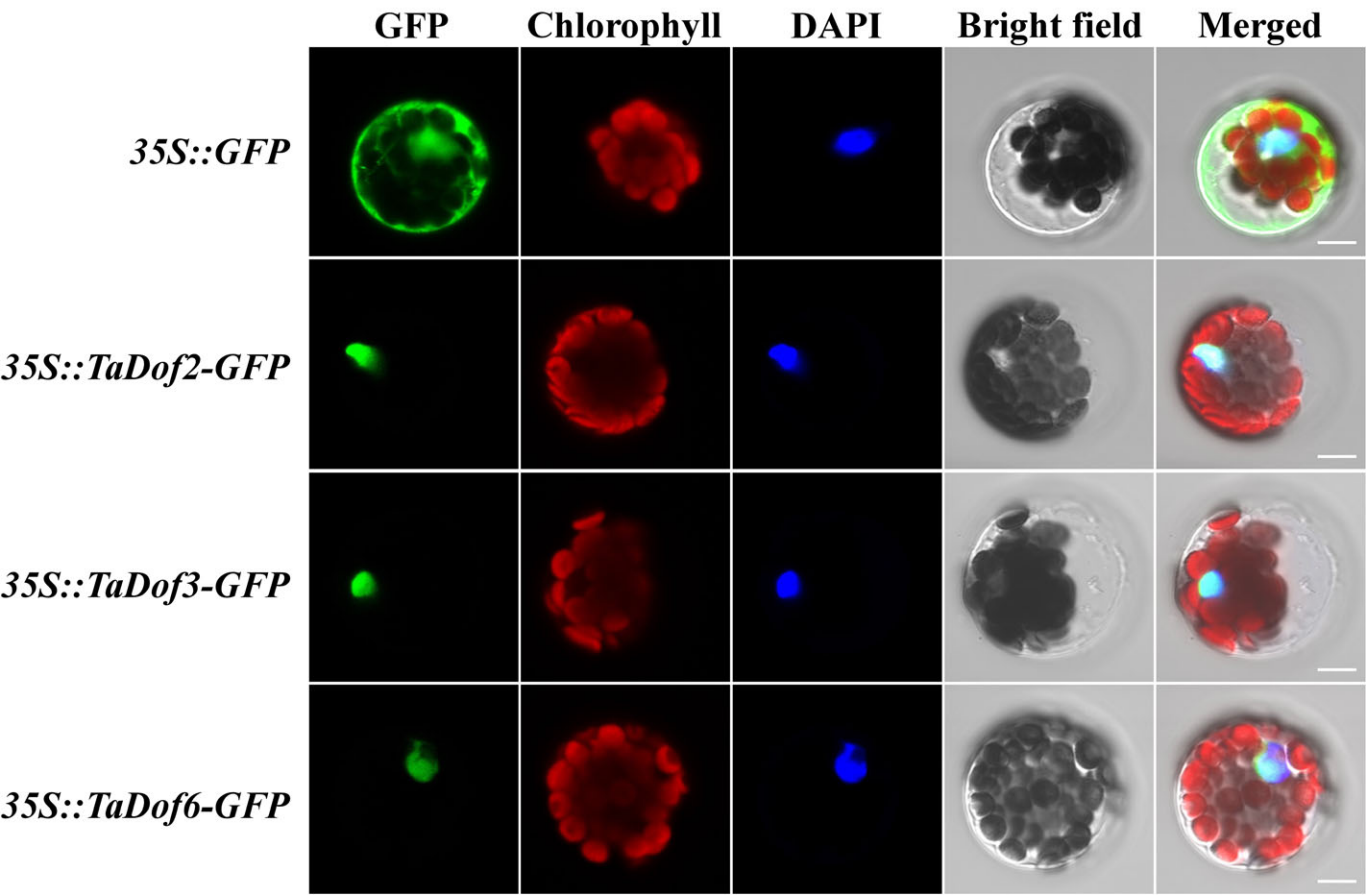
Subcellular localization of wheat TaDof2, TaDof3 and TaDof6. The localization of the nuclei was detected by 4′,6-diamidino-2-phenylindole (DAPI) staining. GFP: GFP fluorescence signal. Green fluorescence indicates the location of TaDofs in the *Arabidopsis* protoplasts; Chlorophyll: chlorophyll autofluorescence signal. Red fluorescent signal indicates the location of chloroplasts in protoplasts; DAPI: Blue fluorescence signal. Blue fluorescence indicates the location of the nucleus stained by DAPI; Bright light: field of bright light; Merged: emergence of the GFP fluorescence signal, chlorophyll autofluorescence signal and bright light field; Nagtive: Wild-type (Clo) *Arabidopsis* protoplast cell. Scale bar = 5 μm

The predicted cellular localizations by the five different software programs showed that all 96 TaDof proteins were located in the nucleus (Table S2). Then three TaDof proteins (TaDof2, TaDof3 and TaDof6) were chosen to further perform transient expression to verify the subcellular localization predictions. The results showed that strong green fluorescent signals of the three GFP fusion proteins were observed in the nucleus (Fig. 2), confirming that these TaDof proteins were located in the nucleus. These results are consistent with the transcription factor characteristics and the software predictions.

### Phylogenetic relationships and molecular characterization of TaDof transcription factors

**Fig. 3:**
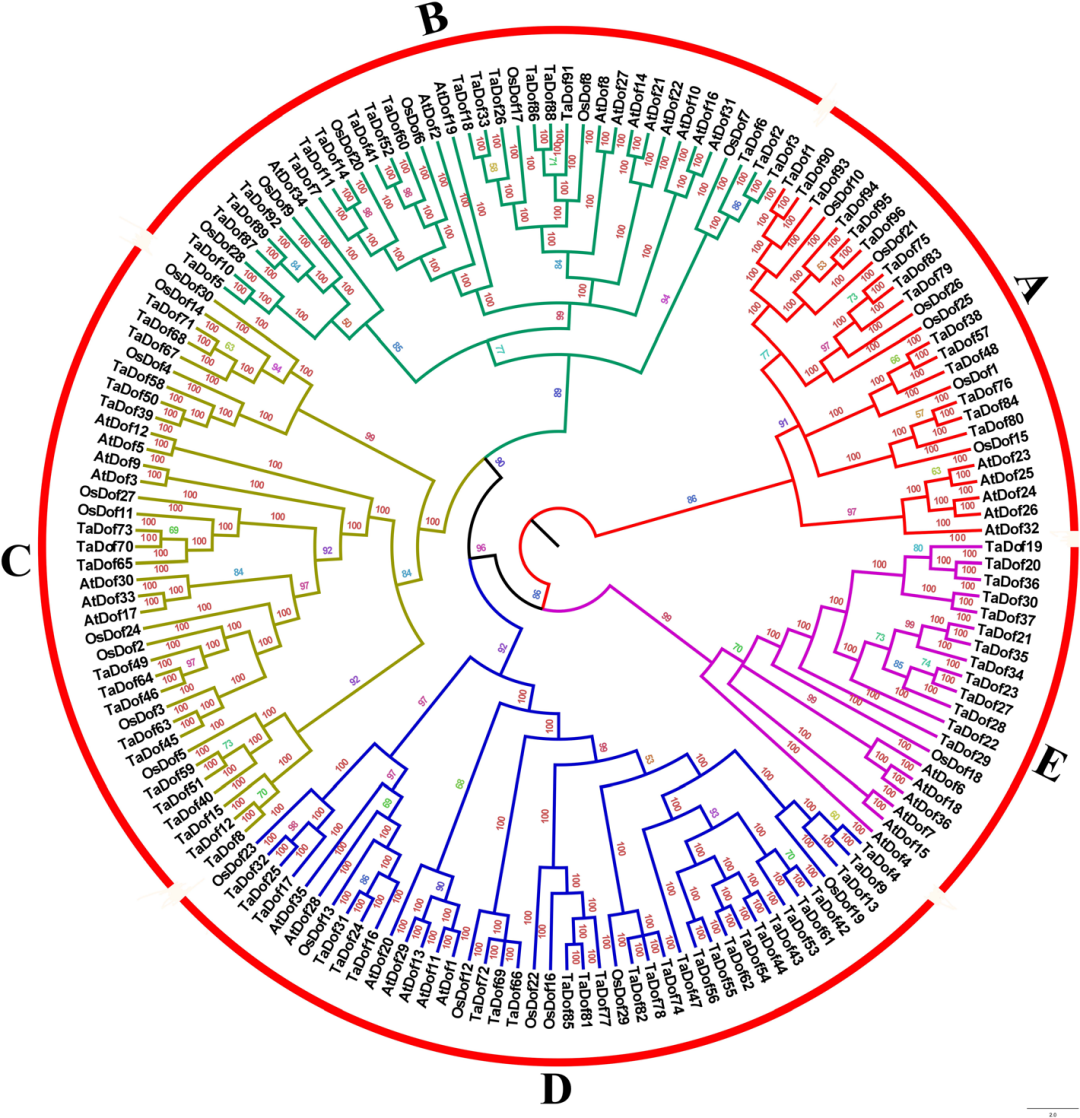
The Bayesian phylogenetic tree of Dof transcription factor gene family from *Triticum aestivum*, *Arabidopsis thaliana* and *Oryza sativa*

**Fig. 4:**
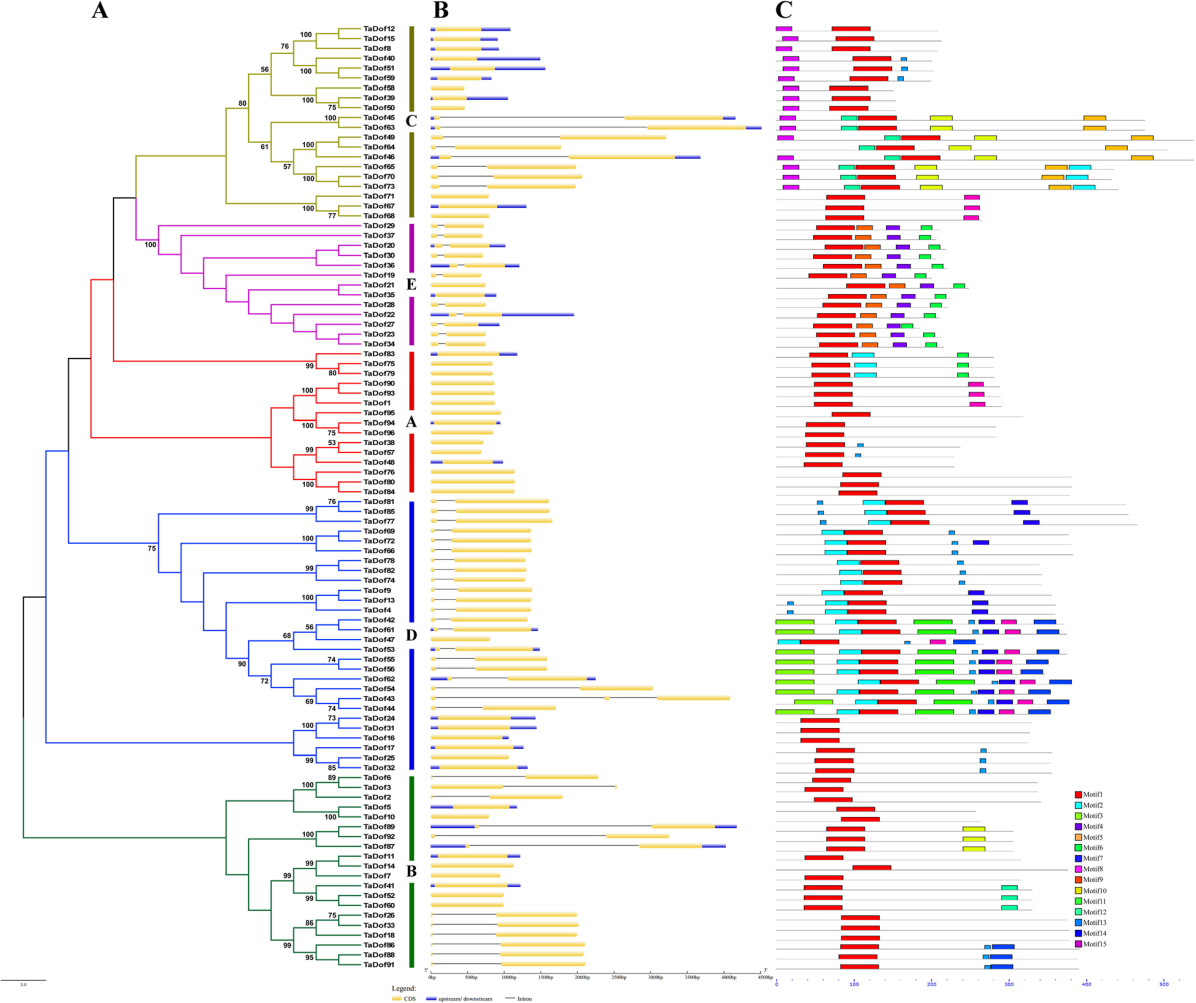
Phylogenetic relationships, exon-intron structures, and motif structures of TaDof gene family members. **a** The neighbor-joining tree of wheat. The color of subclades indicates the five corresponding gene subfamilies. Red, green, yellow, blue and orange represent Group A, Group B, Group C, Group D and Group E, respectively. **b** Exon-intron structures of the *TaDof* genes. Yellow bars: exons; lines: introns; blue bars: 3′ untranslated region. The ratio of bar and line lengths is consistent with that of exons and introns. **c** MEME motif structures. Conserved motifs are indicated in numbered, colored boxes

Multiple sequence alignments of the 162 Dof proteins were performed to construct a Bayesian phylogenetic tree (Fig. 3) and a Neighbor-joining (NJ) phylogenetic tree (Fig. S1). The trees revealed that the 96 *TaDof* genes in wheat were classified into five subfamilies (Group A-E) based on later topology and structural similarity analysis, among which Group D was the largest branch with 28 TaDof members. Both Group B and C had 20 members, followed by Group A with 15 members and Group E with 13 members. As anticipated, the wheat phylogenetic trees constructed by the NJ method (Fig. 4a), maximum likelihood method and minimal evolution method (Fig. S2) showed a similar topological structure for the five subfamilies.

The exon-intron structures of the 96 *TaDof* gene members were analyzed by comparing the CDSs and the complete gene sequences using the GSDSv2.0, and the results are shown in Fig. 4b, including CDS, intron and UTR structures. The number of introns in the *TaDof* genes was extremely small, with 0–2 introns in each gene. Except for *TaDof43* with two introns, 51 *TaDof* genes (53.13%) had only one intron, and the remaining 46 *TaDof* genes (45.83%) had no intron (Table S2). In addition, the members of the same subfamily generally had similar number of introns. The intron length also varied greatly among different subfamilies, likely resulting from the absence or gain of introns during long-term evolutionary processes.

To further investigate the diversity of *Dof* genes in wheat, the MEME program was used to analyze the potential motif composition in the Dof gene family. In total, 15 different motifs were identified (Fig. 4c and Fig. S3). Motif 1, a conserved Dof domain, was uniformly observed in all TaDof proteins. Except for individual members, the same subfamily of Dof proteins generally shared similar motif number, type and spatial arrangement, implying similar functions of Dof proteins in the same subfamily. Group A and B contained fewer motifs, almost exclusively had Motif 1. The specific motif 15 was present in Group C, the conserved motif 2 was present in Group D, and the conserved motifs 4, 6, and 9 occurred in Group E. The remaining motifs 7, 8, 11, 12, 13 and 14 were variable.

### Functional divergence analysis of TaDof transcription factors

The DIVERGE v3.0 software combined with the posterior probability analysis method [42–44] was used to estimate the type-I and type-II functional divergences of the gene group in the Dof family. The results showed that, except for subfamily pairs Group A/Group B, Group A/Group C, Group D/Group C and Group B/Group C, the type-I function divergence coefficient (θ_I_) among other groups ranged from 0.177 to 0.418, which is significantly larger than 0. Among them, likelihood ratio test (LRT) values of the subfamily pairs Group A/Group E, Group C/Group E and Group D/Group E were significantly different (*p* < 0.05), indicating that the possible presence of type-I divergence sites during the evolution between groups of wheat Dof proteins. No significant type-I function divergence was found among other groups. Similarly, the type-II functional divergence coefficient (θ_II_) ranged from − 0.157 to 0.164, indicating that type-II functional divergence sites may also be present (Table 1).

**Table 1:** Functional divergence between subfamilies of the *TaDof* gene family

Group1	Group2	Type I			Type II	
		θ_I_ ± s.e.	LRT	Sites with Qk > 0.8	θ_II_ ± s.e.	Sites with Qk > 0.8
A	B	0.007 ± 0.022	0	None	−0.044 ± 0.128	**30A**
A	C	0.034 ± 0.022	0	None	−0.157 ± 0.157	None
A	D	0.224 ± 0.090	0.596	None	−0.012 ± 0.129	None
A	E	0.418 ± 0.218	0.786	None	−0.007 ± 0.116	52 K, 66 M, 94G, 105D
D	B	0.177 ± 0.022	0	None	−0.048 ± 0.129	71Y
D	C	0.022 ± 0.022	0	None	−0.129 ± 0.163	None
D	E	0.272 ± 0.095	3.989	**30A**	0.053 ± 0.106	**30A**, 32A, 33E, 47E 52 K, 55 N, 66 M, 94G
B	C	−0.053 ± 0.022	0	None	−0.094 ± 0.164	71Y
B	E	0.416 ± 0.125	1.895	None	0.037 ± 0.118	32A, 45 K, 52 K, 55 N, 66 M, 71Y, 75A, 94G
C	E	0.310 ± 0.197	1.115	None	−0.082 ± 0.151	47E

Note: θ_I_ and θ_II_ respectively refer to the coefficients of Type-I and Type- II functional divergence between two groups; LRT, Likelihood Ratio Test; Qk, posterior probability. All sites were locatedon the sequence of TaDof6 according to the results of multiple sequence alignment. The bolted amino acid sites are both Type-I and Type- II functional divergence site

**Fig. 5:**
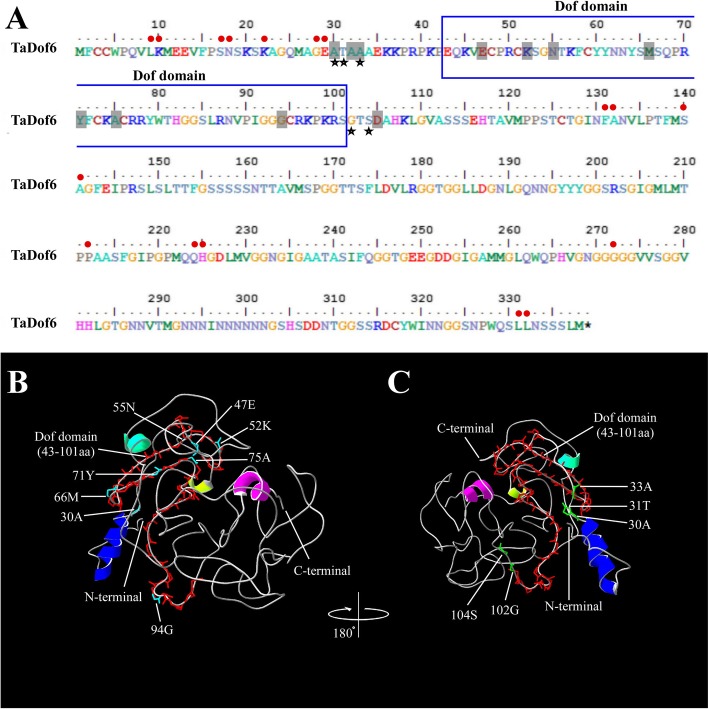
Protein sequences and model building of the 3D structure of TaDof6. **a** Protein sequence of TaDof6. The critical amino acid sites of functional divergence, positive selection and coevolution are labeled respectively with gray shadows, black pentagrams and red circles, respectively. **b** Schematic diagram of 3D structure of TaDof6. The precise positions of seven critical functional divergence sites were colored in light blue. **c** Schematic diagram of 3D structure of TaDof6 obtained by (**b**) rotating 180 degrees anti-clockwise. Five significant positive selection sites are colored in green

Critical amino acid sites were identified in five groups of TaDof subfamilies in the analysis of type-I and type-II functional divergence. In this study, Qk > 0.8 was used as a threshold to screen important amino acid sites, which can reduce the occurrence of false positives. As shown in Table 1, only one type-I functional divergence amino acid site (30A) was detected between Group D and Group E, indicating that the evolutionary rate of this amino acid site might change significantly. Eleven type-II functional divergence sites were found, including 30A, 32A, 33A, 45 K, 47E, 52 K, 55 N, 66 M, 71Y, 75A and 94G. These may be the key amino acid sites affecting physical and chemical properties of TaDof proteins. Apparently, the type-II functional divergence sites were significantly more abundant than type-I functional divergence site, indicating that the type-II functional divergence played a major role in the differentiation of the TaDof gene family. In particular, the amino acid site 30A belonged to both type-I and type-II functional divergence sites, suggesting that the evolutionary rate and physicochemical properties of this site have changed concurrently (Fig. 5a and b).

### Positive selection, coevolution and three-dimensional (3D) structure analysis of TaDofs

The CODEML program in the PAML v4.4 software was used for positive selection analysis and positive selection site identification for the TaDof gene family. The site model used in this study included M0 (one scale) and M3 (discrete) as well as M7 (beta) and M8 (beta and ω) based on the previous method [45]. By comparing M0 and M3 models, we found that the twice log-likelihood difference of the models (2△lnL) was 883.03, indicating that certain amino acid sites might be undergone strong positive selection pressure. Comparison between M7 and M8 models can determine whether *TaDof* gene family members were subjected to positive selection pressure during the evolutionary process. The results revealed that the value of 2△lnL between the two models was 2036.983 with an extremely significant statistical difference. The estimated ω value of the M8 model was 2.55223, which is much higher than 1, indicating that some TaDof amino acid sites were strongly affected by positive selection. In total, 11 positive selectivity amino acid sites were detected in the M8 model, including one significant (102G, *p* < 0.05) and four extremely significant (30A, 31 T, 33A, 104S, *p* < 0.01) positive selection sites (Table 2).

**Table 2:** Tests for positive selection among *TaDofs* gene family using site-specific models

Models	np	Estimates of parameters^a^	lnL	2△lnL	Positively selected sites^b^
M0 (one-ratio)	191	ω = 0.05384	− 4539.929	883.03 (M3 vs M0)	Not allowed
M3 (discrete)	195	p0 = 0.43174, p1 = 0.26150, p2 = 0.30676 ω1 = 0.00039, ω2 = 0.03024, ω3 = 0.23081	− 4098.415		None
M7 (beta)	192	*p* = 0.19976, q = 2.34994	− 4083.224	2036.983 (M8 va M7)	Not allowed
M8 (beta & ω)	194	p0 = 0.99999, *p* = 0.82856, q = 1.22843(p1 = 0.00001), ω = 2.55223	− 5101.716		30A**, 31 T**, 33A**, 45 K, 67S, 82H, 85S, 102G*, 103 T, 104S**, 105D

Note: *, *p* < 0.05 and **, *p* < 0.01 (χ2 test). lnL, log likelihood. 2△lnL, twice the log-likelihood difference of the models. a, ω was estimated under model M0, M3, M7, M8. b, the number of amino acid sites estimated to have undergone positive selection. np, number of free parameters

CAPS, a distance-sensitive coevolutionary analysis software for amino acids, was used to detect the *TaDof* gene family [46], and nine coevolution sites were detected (Table S3). Among them, 8 groups were adjacent in the primary structure, and most of them were distributed in different locations outside the functional structure domain (Fig. 5a).

The 3D structures of TaDof6 proteins constructed by the online software PHYRE2 showed that five significant and extremely significant sites were located on the 3D structure of TaDof6 protein (Fig. 5b-c), which were mainly located at the N-terminal of the Dof protein. These suggest that the N-terminal of TaDof proteins might encounter more positive selection pressures during the evolutionary process.

### Analysis of *cis*-acting elements in wheat Dof transcription factors

The potential *cis*-acting elements in the promoter region among 96 TaDof transcription factor members were analyzed by the online tool PlantCARE, which can benefit the understanding of *TaDof* gene expression and function [47]. In total, seven types of *cis*-acting elements were found in the promoter region of the *TaDof* genes, as shown in Table S4.

Light responsive elements are a very abundant class of *cis*-acting elements among the *TaDof* gene family members, including G-box, Sp1, and Box 4. G-box seems to be the most abundant type of light responsive elements in the *TaDof* gene family, with a cumulative number of 263. Only 17 members of the *TaDof* gene family did not contain G-box while the remaining members had at least one G-box copy. Hormone responsive elements, mainly including TATC-box, GARE-motif, TCA-element, TGA-element, ABRE, TGACG-motif and CGTCA-motif, participate in response to gibberellin, salicylic acid (SA), auxin, abscisic acid (ABA) and methyl jasmonic acid (MEJA). Among them, ABRE (87.5%), TGACG-motif (78.12%) and CGTCA-motif (79.17%) were present in a large number of members, with an average number of copies of 3.12, 2.67 and 2.66, respectively.

Environmental stress-related elements are also noteworthy. For instance, the GC-motif (68 copies) and ARE (108 copies), which are involved in the regulation of gene expression in the absence of oxygen stress, were found to be relatively abundant. Some *TaDof* genes harbored MBS (MYB binding site, which is involved in drought-inducibility), indicating that the expression of these *TaDof* genes can be influenced by drought. And *TaDof* gene family members may be involved in defensive damage recovery response and temperature change response due to the presence of WUN-motif, TC-rich repeats, and LTR elements.

Additionally, CCGTCC-box, CAT-box and O2-site accounted for 33.6, 25.1 and 21.7% of the total number of development related elements, respectively. These elements are involved in the expression and activation of meristematic tissues and the regulation of gliadin metabolism. The promoter-related elements TATA-box and CAAT-box had the largest number of copies per gene, with an average of 13.96 and 15.47, respectively. Except for the promoter of *TaDof86* that had no TATA-box, the other members of the wheat TaDof protein family all contained these two types of *cis*-acting elements related to transcriptional regulation.

In addition, we also counted the number of each *cis*-acting element present in the subfamily. Interestingly, the results showed that the subfamily has a clear preference for the *cis*-acting elements contained in the seven major classes of *cis*-acting elements. For example, for light responsive elements, subfamily A had a relatively large number of A-box element, subfamily D had many Box-4 and TCCC-motif elements. Among the development related elements, subfamily A had the largest number of CCGTCC-box, while subfamily D had 59.3% of the total number of O2-site. For hormone response elements, subfamily A had the most TGACG-motif and CGTCA-motif elements. The number of AREs in the environmental stress-related elements in subfamily D accounted for 51.5% of the total. Particularly, TATA-box and CAAT-box of promoter binding elements were extremely abundant in subfamily D (Table. S5).

### Expression of *TaDof* genes in different organs and developmental stages

**Fig. 6:**
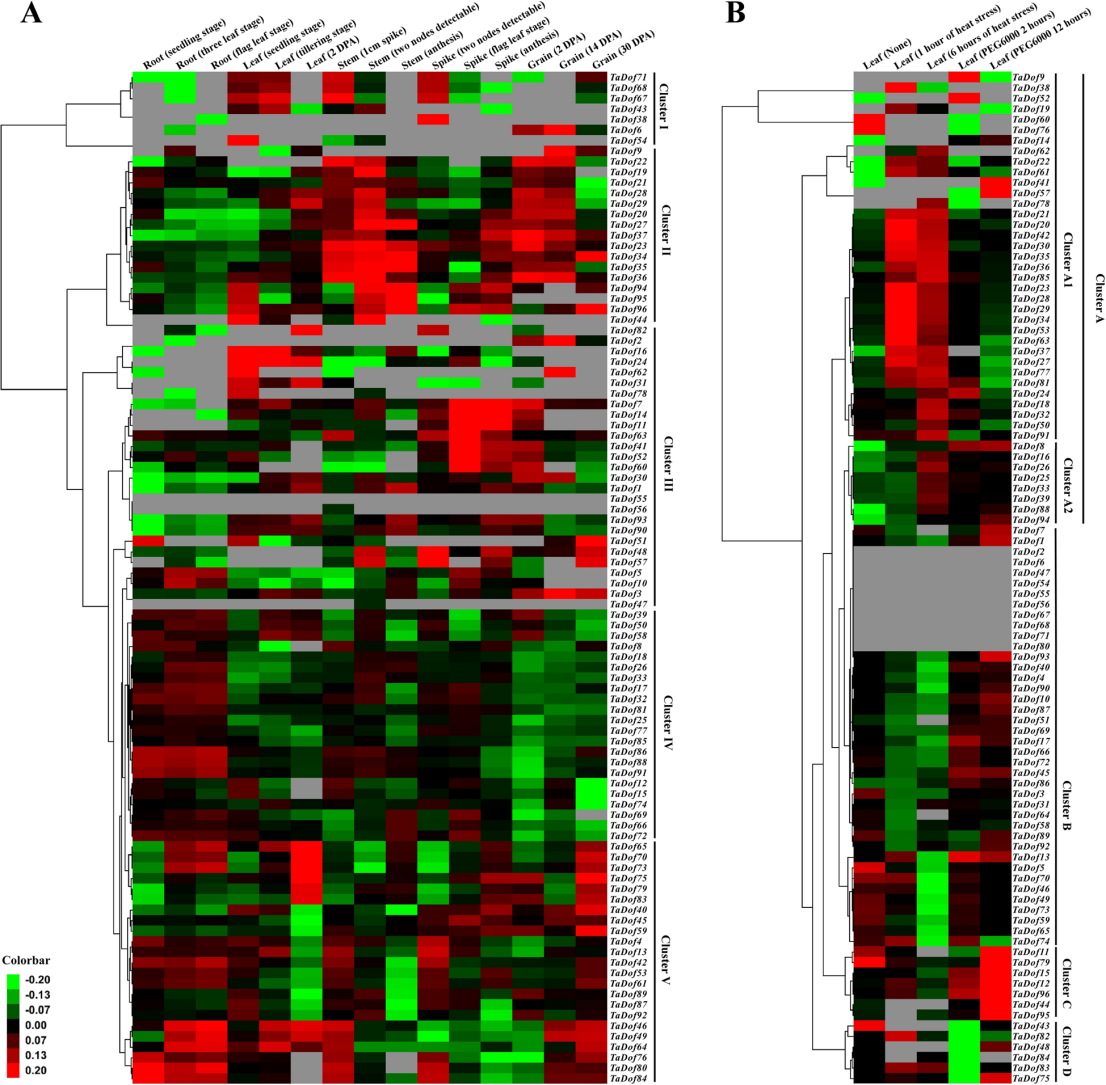
Heat map representation of *TaDof* genes across different tissues and developmental stages and abiotic stresses. **a** Heat map of the expression profiling of wheat *TaDof* genes at seedling, vegetative and reproductive stages. RNA-seq data were obtained from root, leaf, stem, spike and grain of Chinese spring cultivar. **b** Heat map of the expression profiling of wheat *TaDof* genes at seedling under heat or PEG stress. The largest values are displayed as the reddest (hot), the smallest values are displayed as the greenest (cool), and the intermediate values are a lighter color of either red or green. The gray represents the value of 0 in the original RNA-seq data, and the cluster software automatically recognizes this part of the data as “missing”, which was shown in gray. Raw data were normalized by the following equation: reads/kilobase/million

Analysis of RNA-seq data of different organs at different developmental stages found that *TaDof* genes showed different expression patterns in different organs and developmental stages (Fig. 6a and Table S6). In general, the 96 *TaDof* genes could be divided into five groups with distinct expression patterns (Cluster I-V). The seven genes in Cluster I with exhibited a high expression level in leaf, stem and spike, especially at the early developmental stages. However, the expression of some *TaDof* genes, such as *TaDof43*, *TaDof38*, *TaDof6* and *TaDof54,* was either very low or undetectable in certain developmental stages. Cluster II contained 17 *TaDof* genes that were significantly expressed in all stages of the stem growth, and generally with a low expression in root and high expression in seeds at two and 14 days post anthesis (DPA). In Cluster III, 27 *TaDof* genes were significantly expressed in spikes or grains, but some genes, such as *TaDof55,* were not expressed at different growth stages in five organs. In Cluster IV, 22 genes were preferentially expressed in root and their expression levels were relatively low in both leaves and seeds. Cluster V included 23 *TaDof* genes that were significantly expressed at certain periods in all organs, such as *TaDof40*, *TaDof45* and *TaDof59* which were expressed at the late stages of grain development.

It is worth mentioning that most homologous genes had very similar expression patterns during growth and development (Fig. 6a and Fig. 8). Further analysis found that some *TaDof* genes which clustered in the same subfamily of the phylogenetic tree (Fig. 3) generally also had similar expression patterns. For example, except for *TaDof30*, all other members of Group E which clustered in Cluster II were significantly expressed in both stems and seeds. However, some other *TaDof* members, even the homologous genes with highly conserved amino acid sequences in the same subfamily showed distinct expression patterns. For example, 20 *TaDof* genes in Group C displayed four expression patterns (Cluster I/III/VI/V), of which the expression of two pairs of homologous genes *TaDof45*/*TaDof63* and *TaDof51*/*TaDof59* was distinct. Both *TaDof45* and *TaDof59* were clustered in Cluster V and showed higher expression in the spike and grain, while *TaDof63* and *TaDof51* were clustered in Cluster III and had a peak expression in both spike and root. In particular, the homologous genes *TaDof2*, *TaDof3*, and *TaDof6* clustered in a small branch of the phylogenetic tree showed a high expression in the endosperm, but the corresponding expression values detected in the qRT-PCR experiment were particularly low in other tissues (Fig. S4).

### Expression profiling of *TaDof* genes in response to various abiotic stresses

The publicly available RNA-seq data of wheat leaves under polyethylene glycol (PEG)-simulated drought and heat stresses were used to show the expression profile of the *TaDof* genes (Table S6 and Fig. 6b). Since 10 *TaDof* genes lacked RNA-Seq atlas data, only 86 genes were analyzed, which were divided into four distinct expression patterns (Cluster A-D in Fig. 6b). Cluster A could be further divided into cluster A1 and cluster A2, and contained 43 genes (50%). Cluster A1 containing 35 genes was significantly upregulated under heat treatment, and cluster A2 containing 8 genes showed obvious upregulation after heat and PEG treatments. Cluster B had 30 genes (34.9%), and more than half of them were upregulated under PEG stress, but all of them were down-regulated under heat stress. Cluster C included seven *TaDof* genes whose expression was downregulated under heat stress, but upregulated under PEG treatment, particularly at 12 h. Six *TaDof* genes in Cluster D were generally downregulated at the early stages of seedling growth when exposed to PEG stress, but were upregulated at 12 h after PEG treatment.

**Fig. 7:**
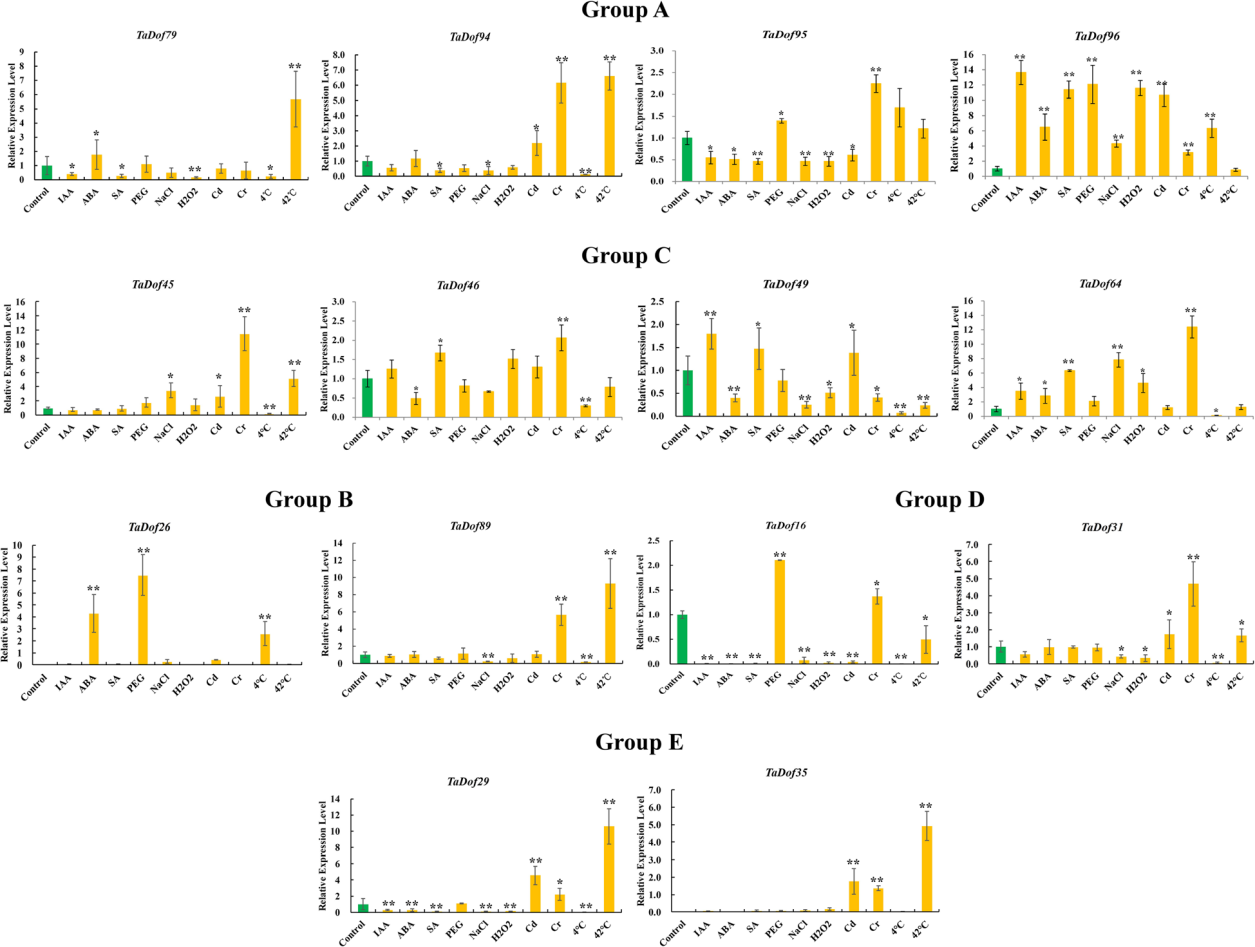
Expression patterns of ten *TaDof* genes from different subfamilies in the seedling leaves of wheat cultivar Zhongmai 175 under various abiotic stresses. The abiotic stress treatments included IAA, ABA, SA, Cr^3+^, Cd^2+^, NaCl, PEG, cold (4 °C) and heat (42 °C). Statistically significant differences between control group and treatment group were calculated by an independent Student’s t-tests: “*” *p* < 0.05; “**” *p* < 0.01

To further validate the expression profile of *TaDof* genes in different organs under various abiotic stresses, we selected 17 representative *TaDof* genes from the five groups for qRT-PCR analysis, and their primer sequences are listed in Table S7. In leaves, 11 genes (*TaDof79*, *TaDof94*, *TaDof95, TaDof96, TaDof45*, *TaDof49*, *TaDof64, TaDof16*, *TaDof31*, *TaDof89* and *TaDof29*) responded to almost all stress treatments evaluated. In particular, three *TaDof* genes displayed a significantly upregulated expression in response to multiple abiotic stresses, including *TaDof96* under all stress except for heat stress, *TaDof26* under ABA, PEG, and cold treatments, and *TaDof35* under Cr^3+^, Cd^2+^, and heat treatments. All *TaDof* genes except *TaDof96* and *TaDof26* were significantly downregulated under cold treatment, whereas all genes, except *TaDof16*, *TaDof26* and *TaDof49,* were significantly upregulated under heat treatment. However, both *TaDof16* and *TaDof26* were significantly upregulated under PEG treatment. These results are generally consistent with the RNA-seq data described above (Table S8). According to the results of RNA-seq datas and qRT-PCR, the expression levels of the most homologous genes in response to abiotic stress were significantly different. For example, *TaDof96* was significantly upregulated under all stress treatments except for heat stress, but the homologous genes (*TaDof94* and *TaDof95*) were downregulated under most stress treatments (Figs. 6b and 7).

In addition, seven *TaDof* genes (*TaDof79*, *TaDof95, TaDof96, TaDof49*, *TaDof64, TaDof16* and *TaDof29*) showed a sensitive response to hormone stress, in which *TaDof*96 and *TaDof* 64 were significantly upregulated under three hormone stresses, *TaDof*49 was significantly upregulated under IAA and SA treatment and the other genes were generally downregulated under hormonal stress. When subjected to heavy metal stress, the expression of most *TaDof* genes was significantly upregulated, especially under Cr^3+^ stress. Additionally, six *TaDof* genes (*TaDof79*, *TaDof95, TaDof49*, *TaDof16*, *TaDof31*, and *TaDof29*) were significantly downregulated after oxidative stress. Under salt stress, seven *TaDof* genes (*TaDof94*, *TaDof95*, *TaDof89*, *TaDof16*, *TaDof49*, *TaDof31* and *TaDof29*) were downregulated, whereas three *TaDof* genes (*TaDof96, TaDof45,* and *TaDof64*) were significantly upregulated (Fig. 7).

**Fig. 8:**
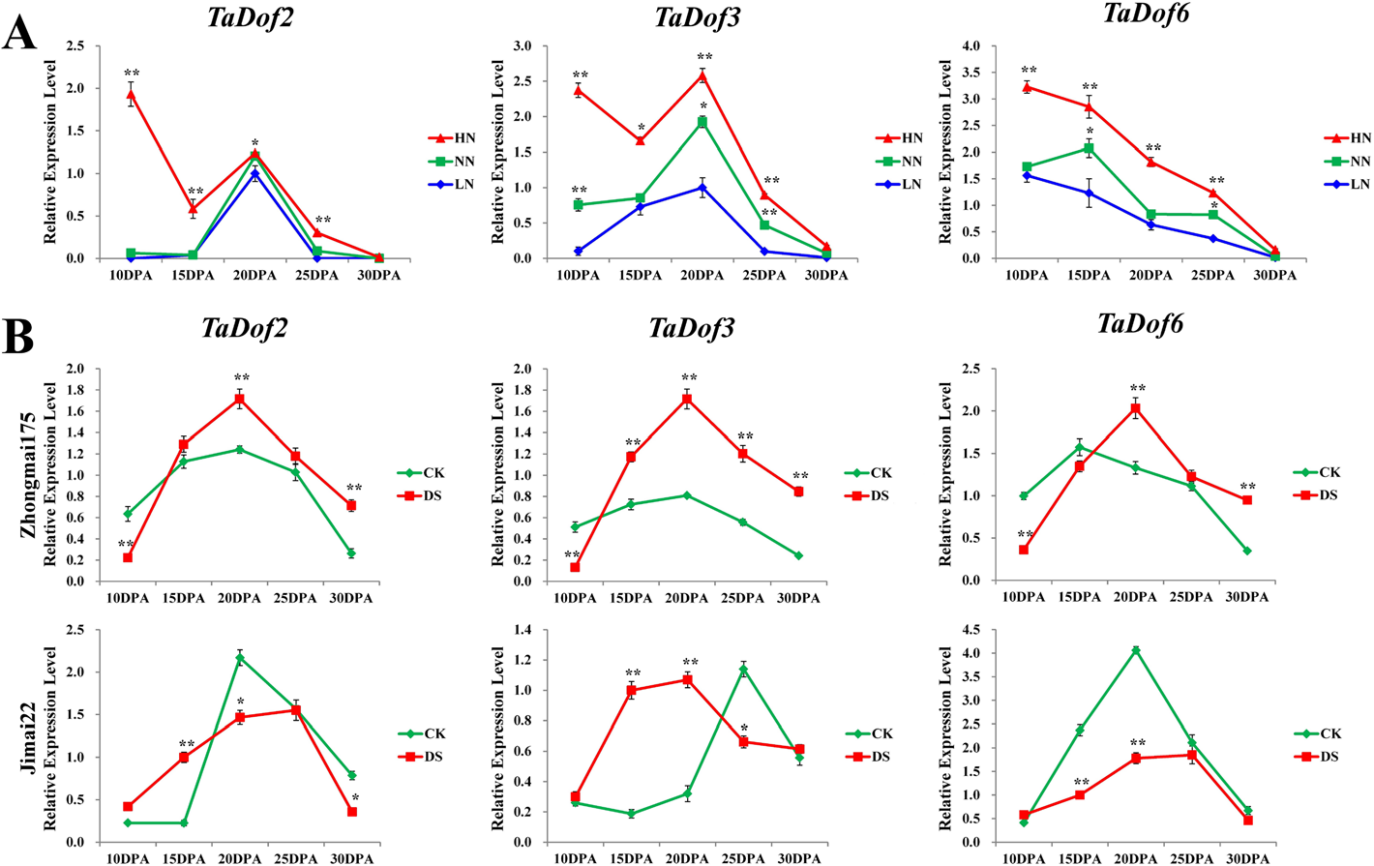
Expression pattern of wheat *TaDof2/3/6* genes. **a** Expression patterns of *TaDof2/3/6* genes during grain development of wheat cultivar Zhongmai 175 and in response to high and low nitrogen treatments. LN (low-nitrogen): without fertilization application after sowing; NN (normal nitrogen): fertilization with 180 kg/hm^2^; HN (high-nitrogen): fertilization with 240 kg/hm^2^. **b** Expression patterns of *TaDof2/3/6* genes during grain development of wheat cultivars Zhongmai 175 and Jimai 22 and in response to drought stress. CK: the control group with two-irrigation at jointing and anthesis stages; DS: drought stress group without irrigation after sowing. Expression of the wheat *Ubiqutin* gene was used as a reference gene. Statistically significant differences between control group and treatment group were calculated by an independent Student’s t-tests: “*” *p* < 0.05; “**” *p* < 0.01

In grains, three highly expressed *TaDof* genes (*TaDof2*, *TaDof3* and *TaDof6*) showed up-and-down expression patterns along with grain development, except for *TaDof6* under nitrogen treatment. Their expression was highly induced and inhibited by high-nitrogen and low-nitrogen treatment, respectively. The effects of nitrogen stress on the expression of the *TaDof3* and *TaDof6* genes were more obvious during grain development (Fig. 8a). Under drought stress, three *TaDof* genes were significantly upregulated in the early developmental stages of the wheat cultivars Zhongmai 175 and Jimai 22 (Fig. 8b).

## Discussion

### Molecular characterization and evolution of wheat Dof transcription factors

According to the cluster analysis results of *A. thaliana* and rice [3, 32], the sorghum Dof gene family was divided into six subfamilies (Group A-F), of which Group B had only four *AtDof* members, namely *AtDof4.2*, *AtDof 4.3*, *AtDof4.4* and *AtDof4.5* [34]. In this study, we used the recently released wheat genome database to identify 96 *Dof* genes at the genome-wide level (Table S2). The 96 *Dof* genes were classified into five subfamilies (Group A-E) using the Bayesian method, in which the four AtDof members were categorized into Group A (Fig. 3), slightly different from sorghum Dof gene classification.

The exon-intron organization can be used as supporting evidence to determine the evolutionary relationships among genes or organisms [48, 49]. In general, the wheat *TaDof* genes in the same subfamily share similar exon-intron structures, but differences are present in different subfamilies. The distribution of motifs among Dof proteins is indicative of evolutionary relationship as deduced by phylogenetic tree [50]. The results shown in Fig. 4 demonstrated that the sequence structure of motif 1 identified by MEME was consistent with the Dof domain, which may be involved in binding to a particular promoter sequence. Meanwhile, at least one or two conservative motif types and spatial arrangements in TaDofs are present in the same subfamily, but obvious differences occur between different subfamilies, implying certain functional similarities of Dof members within the same subfamily. In addition, the structural conservation of the *TaDof* genes in subfamilies was consistent with other plants such as *Arabidopsis* and rice [32], barley [33], sorghum [34] and *B. distachyon* [35].

Wheat genomes (BBAADD) consist of three related sub-genomes, and they were derived from three different diploid species, respectively [51]. A total of 96 *TaDofs* transcription factor family members were identified in this study. They were unequally distributed in sub-genomes A, B, and D, with 34, 32, and 30 members, respectively (Fig. 1). This suggests that there may be a loss of homologous genes during evolution. On the other hand, the retained genes and their distribution on chromosomes provided valuable reference for the polyploidization of wheat chromosomes. It is worth noting that the *TaDof* gene on chromosome 4A was opposite to the distribution of homologous genes on chromosomes 4B and 4D, basically consistent with the recent research [52]. This indicates that the chromosome 4A arm had been reversed during the evolution of hexaploid wheat [53]. We also found that the genes located at the lower part of chromosomes 2 and 3 were closely arranged, and their sequences were highly consistent. They meet the definition of tandem duplication, so they are considered as tandem duplication genes formed during long-term evolution. Interestingly, although the sequences of *TaDof47* and *TaDof42–44* are highly similar, there are too many genes between them to consider as a tandem duplication gene. Moreover, it didn’t meet the requirements of segmental duplication gene, so we speculate that it may be caused by the insertion of a short chromosome segmental between *TaDof44* and *TaDof47* during the evolution. The functional diversity of proteins is generally resulted from gene duplication events or the formation of new species [54]. According to our results, type-II functional divergence sites were significantly more abundant than type-I functional divergence sites (Table 1), demonstrating the important roles of the physical and chemical property changes of amino acids in the diversification among the subfamilies. All seven type-II functional divergence sites (47E, 52 K, 55 N, 66 M, 71Y, 75A and 94G) are located in the Dof domain (Fig. 5), indicating that they may be the main driving force promoting the variation of the Dof domain and the functional divergence of the wheat Dof family. At the molecular level, the amino acid mutations that increase adaptation to the environment were retained through positive selection [55]. Five sites (30A, 31 T, 33A, 102G and 104S) were found to have been subjected to strong positive selection, and may play important roles in functional divergence. Meanwhile, complex coevolutionary networks play important roles in the stability of protein structure and function during evolutionary process [46]. Nine coevolutionary amino acid sites detected in TaDof family members (Table S3) may play roles in maintaining the spatial structure of Dof proteins. These coevolutionary sites may not only perform an important function, but also play a key role in the evolution of the wheat Dof gene family. Therefore, these special amino acid sites might provide useful information for further deciphering the functional attributes of *TaDof* genes.

### Potential roles of *TaDof* genes in tissue differentiation and organ development

Phylogenetic and expression analyses can provide important clues about the potential functions of wheat *Dof* genes. As reported in other plants [36, 56, 57], the *TaDof* genes showed a specific and preferential expression in different organs and developmental stages (Fig. 6a), implying their involvement in plant growth and development. Phylogenetic analysis revealed that eight *TaDof* genes (*TaDof45/46/49/63/64/65/70/73*) showed a high sequence similarity to the five *A. thaliana Cycling DOF Factors* (*CDF1–5*) in the Group C subfamily, and they were expressed in the developing grains and stem/spike at the flowering stage (Fig. 3). *CDF1* represses the transcription of Constans (*CO)* and thereby represses flowering in *A. thaliana* [58]. Thus, it is likely that these *TaDof* genes also play roles in spike and seed development.

The homologous *TaDof2*, *TaDof3* and *TaDof6* genes were highly expressed in the early and middle stages of grain development (Fig. 6a). They were closely clustered with *RPBF* (*OsDof7*) in the phylogenetic tree (Fig. 3), suggesting their functional similarity. The previous study showed that *RPBF* gene, predominantly expressed in the maturing endosperm and coordinately expressed with seed storage protein genes, also participated in the quantitative regulation of genes expressed in the endosperm through cooperating with RISBZ1 [19]. Wheat WPBF can trans-activate the native alpha-gliadin gene promoter via interaction with the 5′-TGTAAAG-3′ motif [28]. As PBF homologues, the proteins encoded by the *TaDof2*, *TaDof3* and *TaDof6* genes activate wheat prolamin gene expression during seed development [26, 27]. Furthermore, the promoter activities of WPBF were observed in the vascular system and the seeds of transgenic *A. thaliana*, suggesting that WPBF functions not only in seed development, but also during other plant growth and developmental stages [28]. We found that some development-related elements present in *TaDof2/3/6* are involved in seed-specific regulation (O2-site, RY-element) and meristem expression (CAT-box) or specific activation (CCGTCC-box) (Table S4), which could play important roles in regulating plant growth and seed development.

It is well established that stems contained abundant vascular tissue. The *TaDof* genes in Group E showed predominantly higher transcript levels in stem (Fig. 6), suggesting they may play a role in vascular tissue development. *A. thaliana* Dof factor OBP1 (OBF-binding factor-1) is implicated in a more general control of cell division, and plays an important role in cell cycle re-entry, acting as a transcriptional regulator of key cell cycle genes [59]. Promoter activity analysis suggested that *AtDof5.8* may function in the primary processes of leaf vasculature formation. *AtDof5.8* promoter sequences also contain *cis*-elements for the stage-specific expression during vascular development [60]. In this study, we detected a large number of the meristem-specific activation-related element CCGTCC-box in the *TaDof* gene promoter regions of Group E, and each gene contained more than 2.3 copies (Table S5), indicating that these *TaDof* genes may also function in various vascular development processes.

### Expression and functions of *TaDof* genes in response to abiotic stresses

Dof transcription factors are involved in various abiotic stress responses through the regulation of multiple metabolic pathways. Dof proteins are implicated in responses to plant hormones, such as auxins [61] and gibberellins [8]. When a stressor causes an increase in auxin, the plant activates ABA and other pathways, ultimately promoting the expression of stress-defense genes [62, 63]. The *StDof* genes in potato showed either ABA-dependent or ABA-independent expression pattern [64]. We detected a numerous phytohormone regulation-related elements in the promoter region of the 96 TaDof family members, such as the TCA-element, GARE-motif, TGA-element, ABRE and CGTCA-motif (Table S4). qRT-PCR analysis also revealed that the expression of *TaDof* genes can be induced by IAA, ABA and SA (Fig. 7), indicating that these *cis*-elements related to the phytohormone response play important roles in plant hormone pathways.

Reactive oxygen species (ROS) in plants remain at a relatively stable level under normal physiological conditions. However, heavy metal stress will cause an imbalance in the production and clearance of H_2_O_2_, leading to oxidative damage to plant cells due to excessive accumulation of ROS [65, 66]. We found that eight *TaDof* genes (*TaDof95, TaDof96, TaDof46, TaDof49*, *TaDof64, TaDof16*, *TaDof31* and *TaDof29*) were significantly upregulated under heavy metal stress (Fig. 7). We speculate that *TaDof* genes can act as important regulators in the dynamic regulation of ROS clearance pathways.

The *AtDof* gene *CDF3* is highly induced by drought, extreme environment as well as ABA. Studies showed that *cdf3–1*, a *CDF3* T-DNA insertion mutant, is much more sensitive to drought and low temperature stresses, whereas *CDF3* overexpression promotes the tolerance to drought, cold and osmotic stress [12, 17]. Five homologues of *A. thaliana* CDFs (*SlCDF1–5*) were reported to be differentially induced in response to osmotic, salt, heat, and low-temperature stresses [25]. Most of the *Dof* genes in Chinese cabbage were also quickly upregulated by salt, drought, heat, and cold stress treatments [56]. Furthermore, the *Dof14–15* and *Dof1* genes in wheat were significantly upregulated under drought and salt stresses [27]. In this study, most *TaDof* genes were upregulated under heat stress, and the RNA-Seq data also revealed that the majority of the *TaDof* genes were responsive to heat and PEG stresses (Fig. 6). In particular, the *TaDof16*, *TaDof26* and *TaDof96* genes were upregulated two-, seven- and twelve fold after PEG stress, respectively (Fig. 7). A large number of elements related to environmental stress were also present in the promoter region of *TaDof* genes such as LTR and MBS, and each member had more than 1.2 copies of ARE, GC-motif and WUN-motif (Table S4), which may play an important role in responding to various abiotic stresses.

Introduction of the maize *ZmDof1* gene into rice can increase nitrogen assimilation and enhance plant growth under low-nitrogen conditions [67]. Dof1 and GS work together to regulate the nitrogen metabolism pathway in plants, and to enhance nitrogen assimilation in transgenic tobacco plants grown under low-nitrogen conditions [68]. The *TaDof2*, *TaDof3* and *TaDof6* genes were highly expressed during grain development under drought stress, which improved the synthesis of storage proteins and gluten quality [30]. Consistent with previous studies, in this study, the expression of the *TaDof*2, *TaDof*3 and *TaDof*6 genes was upregulated under drought and nitrogen stresses (Fig. 8). These results suggest the potential roles of *TaDof* genes in the response to abiotic stresses.

## Conclusions

A total of 96 *TaDof* transcription factor genes were identified from the wheat genome database, which were classified into five subfamilies (Group A-E). The members of the TaDof family almost had no introns and all contained a conserved Dof domain. Type-II function divergence was identified as the main reason for the functional diversification of TaDof transcription factors. The *Dof* gene family underwent different degrees of positive selection pressure in the evolutionary process. The nine coevolutionary sites were identified, which may play important roles in maintaining the structural and functional stability of TaDof proteins. Depending on the type and number of *cis*-acting elements, the TaDof genes may be regulated by a variety of hormones and environmental factors. RNA-seq data analysis found that *TaDof* genes showed multiple expression patterns, with obvious expression specificity and preference in different organs and developmental stages, suggesting their potential roles in tissue differentiation and organ development. qRT-PCR analysis further showed that some *TaDof* genes were significantly upregulated in response to single and multiple abiotic stressors, suggesting that they are involved in stress resistance. Our study provides valuable information for further understanding the molecular evolutionary mechanism and functional traits of the plant Dof gene family.

## Methods

### Identification of Dof transcription factors in wheat

The amino acid sequences of 36 and 30 Dof transcription factors of *Arabidopsis thaliana* and *Oryza sativa* were firstly downloaded from the plant transcription factor database (PlantTFDB v4.0, http://planttfdb.cbi.pku.edu.cn/) [32, 69]. Then the obtained Dofs protein sequences of rice and *Arabidopsis* were used for BLASTP operation (E-value ≤1e-5) to retrieve possible TaDofs in wheat from the published common wheat genome database (IWGSC RefSeq v1.0) in Ensemble Plants (http://plants.ensembl.org/). The SMART (http://smart.embl-heidelberg.de/) [70] and Pfam (http://pfam.xfam.org/) [71] websites were used to further verify if the identified proteins had a conserved Dof domain.

### Chromosome location and gene duplication analysis of *TaDof* genes

The chromosome location of *TaDof* genes was obtained from the IWGSC database and visualized by the MapInspect software. The duplication gene pairs in the *Dof* gene family were identified based on the criteria of the previous studies [52, 72]. For segmental duplication analysis, we used a pair of non-homologous but similar genes as the center to take 10 genes from the upstream and downstream respectively to compare the similarity of these genes. Based on the similarity and arrangement of the genes on the chromosomes near the two genes, it was determined whether the two genes were segment duplicate genes. The tandem duplicate analysis of *TaDof* genes was performed according to the method described by Yin et al. [73].

### Subcellular localization of TaDof proteins

The prediction and experimental verification of subcellular localization of TaDofs were performed according to the website and experimental method described by Liu et al. [74].

### Phylogenetic analysis

Multiple sequence alignment of the amino acid sequences of Dof proteins were performed based on MUSCLE program (http://www.ebi.ac.uk/Tools/msa/muscle/) [75, 76]. Subsequently, the Bayesian and N-J phylogenetic trees were constructed based on the method by Han et al. [77]. Furthermore, maximum likelihood and minimal evolution methods were applied for the tree construction to validate the results of the N-J method in MEGA5.0.

### Sequence characteristics analysis

The PlantCARE (http://bioinformatics.psb.ugent.be/webtools/plantcare/html/) was used to analyze *cis*-acting elements in the 1500 bp promoter region upstream of the *TaDofs* [47]. The exon-intron structures map of *TaDofs* were detected by comparing the coding sequences (CDS) and genomic sequences in Gene Structure Display Server v2.0 website (GSDS, http://gsds.cbi.pku.edu.cn/) [78]. Conserved motifs of the TaDof protein sequences were detected by MEME website (http://meme-suite.org/tools/meme), the maximum number of motifs was set to 15 and the remaining operating parameters were unchanged [79, 80]. Theoretical pI/MW of TaDofs was calculated by the Compute pI/MW tool (http://web.expasy.org/compute_pi/).

### Molecular evolution analysis of the TaDofs gene family

DIVERGE 3.0 software package was used to detect the presence of type I and type II functional divergence sites in different subfamilies, and the critical value of posterior probability (Qk) was set to 0.8 [43]. The positive selection analysis of the Dof protein family was performed using site model in the PAML4.4 software package [45, 81, 82]. Coevolution sites between amino acids were identified using Coevolution Analysis using Protein Sequences (CAPS) in PERL software [46, 83].

### Three-dimensional (3D) structure analysis of TaDof proteins

The 3D structures of TaDof proteins were constructed using PHYRE2 website (http://www.sbg.bio.ic.ac.uk/phyre2/html/page.cgi?id=index) [84], and visualized by Pymol software (http://pymol.org/).

### *TaDof* gene expression analysis by RNA-seq data

The RNA-seq data of the *TaDof* genes were downloaded from the expVIP website (http://www.wheat-expression.com/) [85], and cluster analysis was performed by Cluster 3.0 and TreeView software.

### Plant material and abiotic stress treatments

The Chinese wheat cultivar Zhongmai 175 seedlings were cultivated into two leaves and one heart stage according to the culture conditions of Han et al. [77]. Then seedlings were treated with the following conditions: heavy metal stress with 300 μM CrCl_3_ and CdCl_2_, drought stress with 20% (w/v) PEG 6000, salinity stress with 200 mM NaCl, oxidative stress with 15 mM H_2_O_2_, hormone stress with 100 μM ABA (abscisic acid) and SA (salicylic acid), and 10 μM IAA (indole-3-acetic acid), cold stress under 4 °C) and heat stress under 42 °C. The samples from heat stress were collected at 2 h, and other treated seedlings were harvested at 12 h. Three biological replicates were set up for each sample, which were quickly frozen in liquid nitrogen and stored at − 80 °C refrigerator.

Meanwhile, two elite Chinese wheat cultivars Zhongmai 175 and Jimai 22 were planted at the experimental station of China Agricultural University, Wuqiao, Hebei Province (116°37′23″E and 37°16′02″N) during the 2016–2018 wheat growing season. Field experiments included two-irrigation at jointing and anthesis stages for control group (CK), no-irrigation after sowing for drought stress treatment (DS), low-nitrogen (LN) without fertilization application after sowing, normal nitrogen (NN) fertilization with 180 kg/hm^2^ and high-nitrogen (HN) fertilization with 240 kg/hm^2^. Each treatment contained three biological replicates and each plot had 20 m^2^. Plants were marked after flowering, and grain samples from five developmental stages of 10, 15, 20, 25 and 30 days post-anthesis (DPA) were harvested. All samples were quickly collected and immediately frozen in liquid nitrogen, and then stored at − 80 °C prior to analysis.

### qRT-PCR analysis

Total RNA extraction, primer design, qRT-PCR procedures and data analysis were performed according to Cao et al. [86], and three biological replicates were used for each sample. Ubiquitin gene was used as the reference gene. The expression levels of the target genes were determined according to the expression changes relative to the reference gene. At the beginning of the experiment, the relative stability of the reference gene was tested by using various organs and wheat materials treated with different abiotic stress treatments.

## Supplementary information

**Additional file 1:** **Figure S1.** The Neighbour-Joining tree of Dof transcription factor gene family from *Triticum aestivum* L., *Arabidopsis thaliana* and *Oryza sativa* L.

**Additional file 2:** **Figure S2.** The phylogenetic tree of Dof transcription factor gene family from *Triticum aestivum* L. (A) Minimun Evolution Tree. (B) Maximun Likelihood tree.

**Additional file 3:** **Figure S3.** Motifs of TaDof proteins.

**Additional file 4:** **Figure S4.** Expression profiling of *TaDof2*, *TaDof3* and *TaDof6* genes in seven wheat tissues and organs.

**Additional file 5:** **Table S1.** The nomenclature, characteristics of *Dof* genes and their deduced proteins in *Arabidopsis thaliana* and *Oryza sativa subsp. japonica.* The data (accession numbers and amino acid sequences) of Dof transcription factor for *Arabidopsis* and rice are obtained from the Plant Transcription Factor Database (http://planttfdb.cbi.pku.edu.cn/family.php?sp=Ath&fam=Dof; http://planttfdb.cbi.pku.edu.cn/family.php?sp=Osj&fam=Dof). **Table S2.** The nomenclature, characteristics of *Dof* genes and their deduced proteins in *Triticum aestivum* L. The accession numbers and sequences (genomic, amino acid, and CDS sequences) of the 96 wheat Dof genes were obtained from *Triticum aestivum* genome data (IWGSC) in Ensemble Plants (http://plants.ensembl.org/Triticum_aestivum/Info/Index). **Table S3.** Coevolution sites in TaDofs. **Table S4.** *Cis*-element analysis of 1500 bp nucleotide sequences data upstream of the translation initiation codon of *TaDof* genes. **Table S5.** The number of each *cis*-acting element contained in the subfamily. **Table S6.** The RNA-Seq atlas data of the *TaDof* genes. **Table S7.** Primers used for qRT-PCR of *Dof* genes in wheat. **Table S8.** The relative expression values in qRT-PCR and RNA-Seq atlas data of *TaDofs* under abiotic stresses. (A) The relative expression values of *TaDofs* in qRT-PCR under abiotic stresses. (B) The RNA-Seq atlas data of *TaDofs* under abiotic stresses.

## Data Availability

The 96 wheat Dof genes data and their accession numbers listed in Table S2 were derived from *Triticum aestivum* genome data (IWGSC) in Ensemble Plants (http://plants.ensembl.org/Triticum_aestivum/Info/Index), and all accession numbers allow access to public data. The wheat genome sequencing data access URL in Ensemble Plants is ftp://ftp.ensemblgenomes.org/pub/release-46/plants/fasta/triticum_aestivum/dna/. The data (accession numbers and amino acid sequences) of Dof transcription factor for *Arabidopsis* and rice are obtained from the Plant Transcription Factor Database (http://planttfdb.cbi.pku.edu.cn/family.php?sp=Ath&fam=Dof; http://planttfdb.cbi.pku.edu.cn/family.php?sp=Osj&fam=Dof). The RNA-seq data of the 96 wheat Dof genes with specific accession numbers from Ensemble Plants (http://plants.ensembl.org/Triticum_aestivum/Info/Index) are available on the expVIP website (http://www.wheat-expression.com/), and the transcriptome data of hexaploid wheat tissues, cultivars and stress conditions access URL in expVIP website is https://opendata.earlham.ac.uk/wheat/under_license/toronto/Ramirez-Gonzalez_etal_2018-06025-Transcriptome-Landscape/expvip/RefSeq_1.1/ByTranscript/. All data generated or analyzed during this study are included in this published article and its supplementary information files.

## Abbreviations

ABA: Auxin, abscisic acid
CDF: Cycling DOF Factors
CDS: Coding sequence
CK: Control group
Dof: DNA binding with one finger
DPA: Days post anthesis
DS: Drought stress treatment
GFP: Green fluorescent protein
GSDS: Gene structure display server
HN: High-nitrogen
IAA: Indole-3-acetic acid
IWGSC: International Wheat Genome Sequencing Consortium
LN: Low-nitrogen
LRT: Likelihood ratio test
MBS: MYB binding site
MCMC: Markov Chain Monte Carlo
MEJA: Methyl jasmonic acid
NJ: Neighbor-joining
NN: Normal nitrogen
OBP: OBF-binding factor
PBF: Prolamin-box binding factor
PEG: Polyethylene glycol
PlantTFDB: Plant Transcription Factor Database
RPBF: Rice PBF
SA: Salicylic acid
3D: Three-dimensional
TFs: Transcription factors
