# Supplementary figures and images for: Genome-wide analysis of wheat DNA-binding with one finger (Dof) transcription factor genes: evolutionary characteristics and diverse abiotic stress responses
Source: BMC Genomics. 2020 Apr 3;21:276. doi: 10.1186/s12864-020-6691-0 (PMC7118883; doi:10.1186/s12864-020-6691-0)

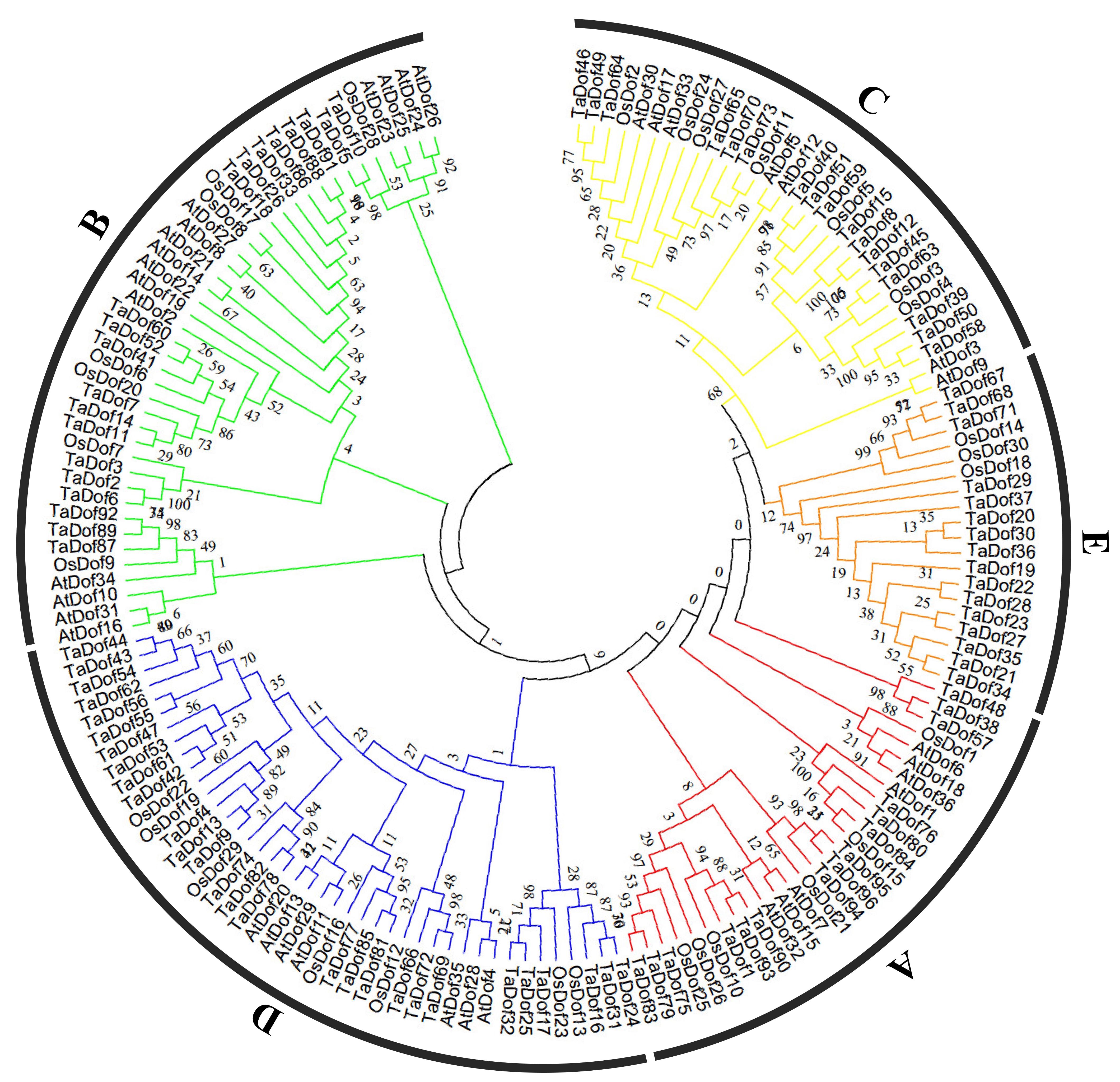

Supplement: Supplementary file 1 — Additional file 1: Figure S1. The Neighbour-Joining tree of Dof transcription factor gene family from Triticum aestivum L., Arabidopsis thaliana and Oryza sativa L. [file 12864_2020_6691_MOESM1_ESM.jpg]

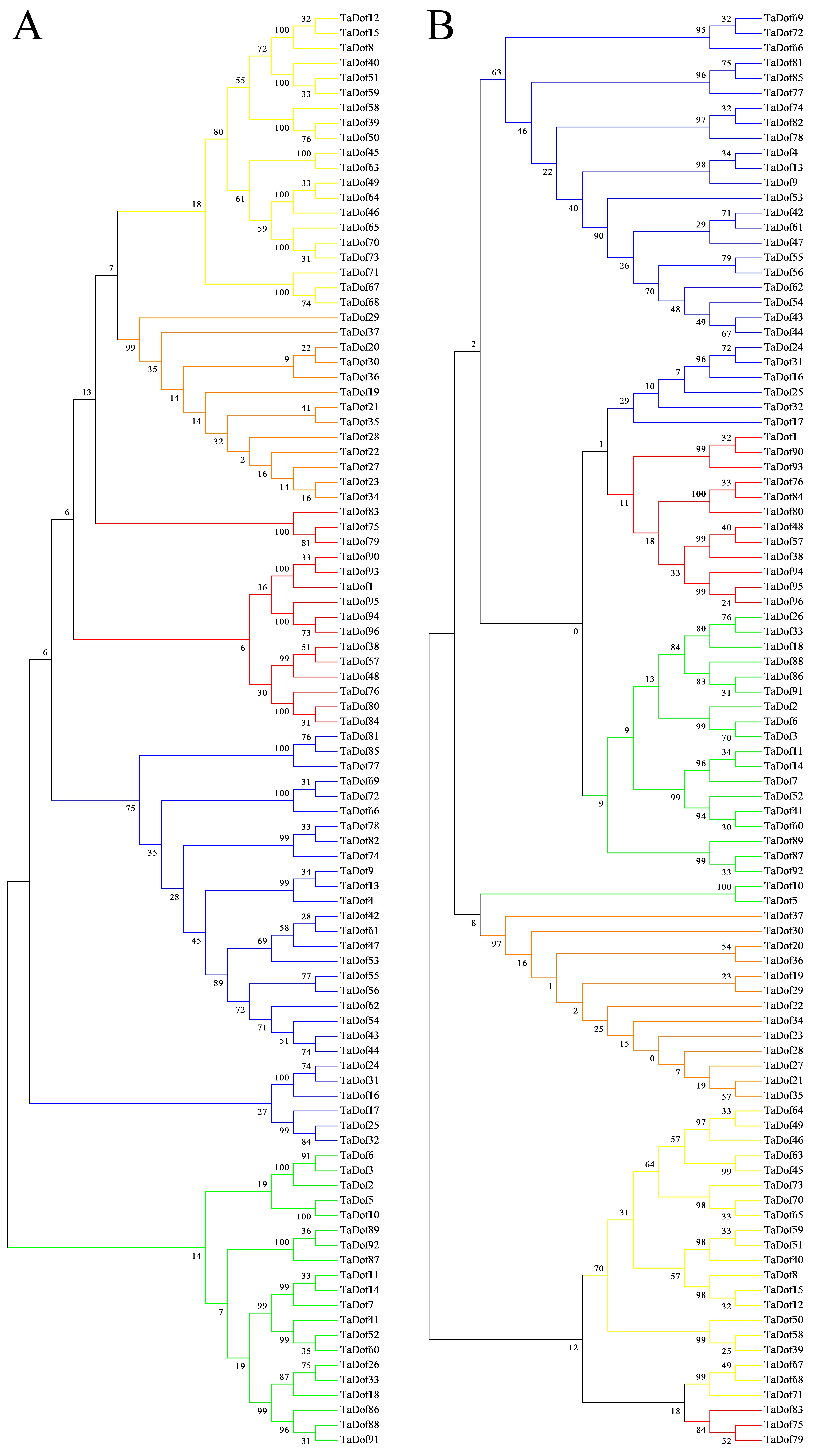

Supplement: Supplementary file 2 — Additional file 2: Figure S2. The phylogenetic tree of Dof transcription factor gene family from Triticum aestivum L. (A) Minimun Evolution Tree. (B) Maximun Likelihood tree. [file 12864_2020_6691_MOESM2_ESM.jpg]

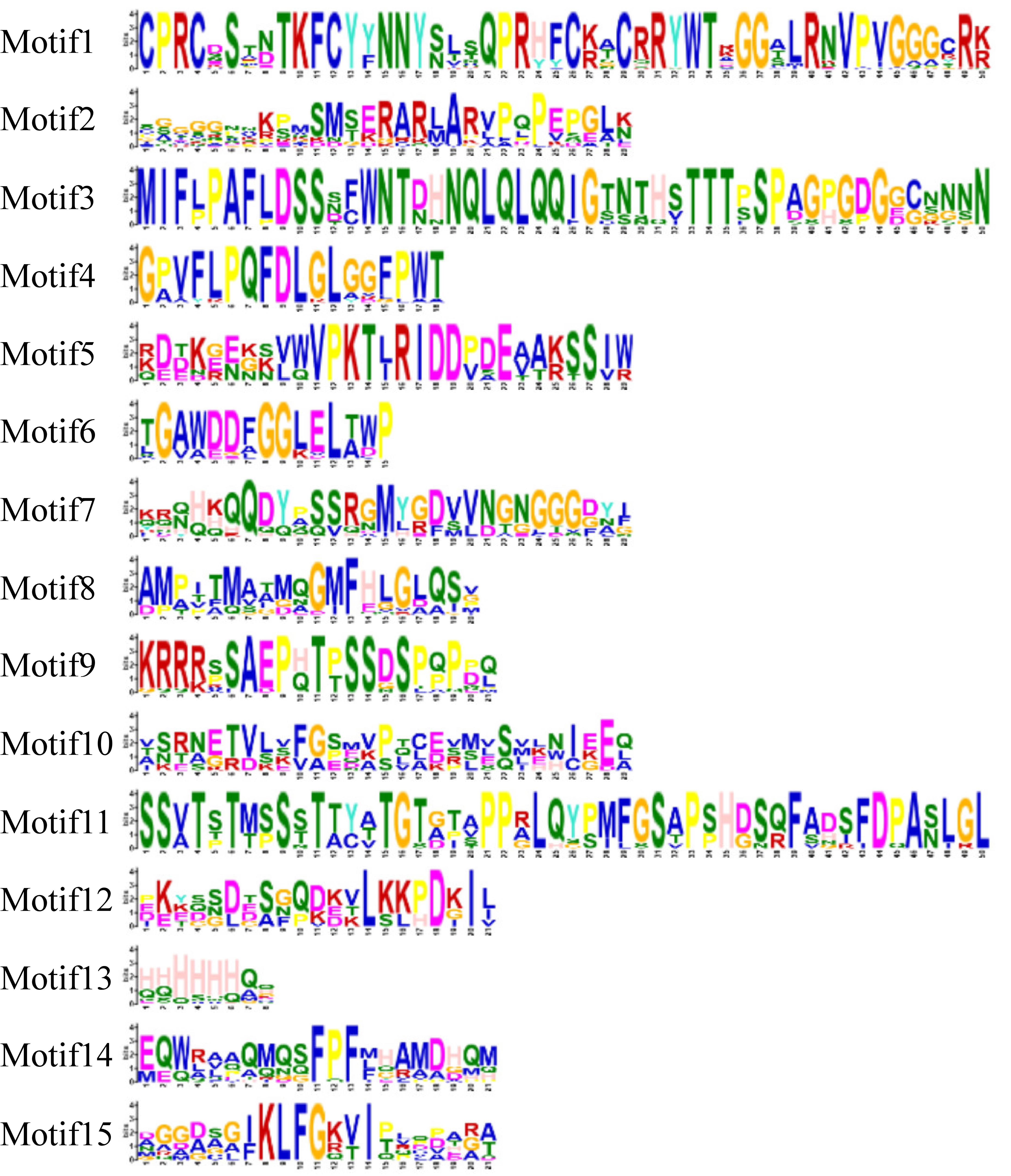

Supplement: Supplementary file 3 — Additional file 3: Figure S3. Motifs of TaDof proteins. [file 12864_2020_6691_MOESM3_ESM.jpg]

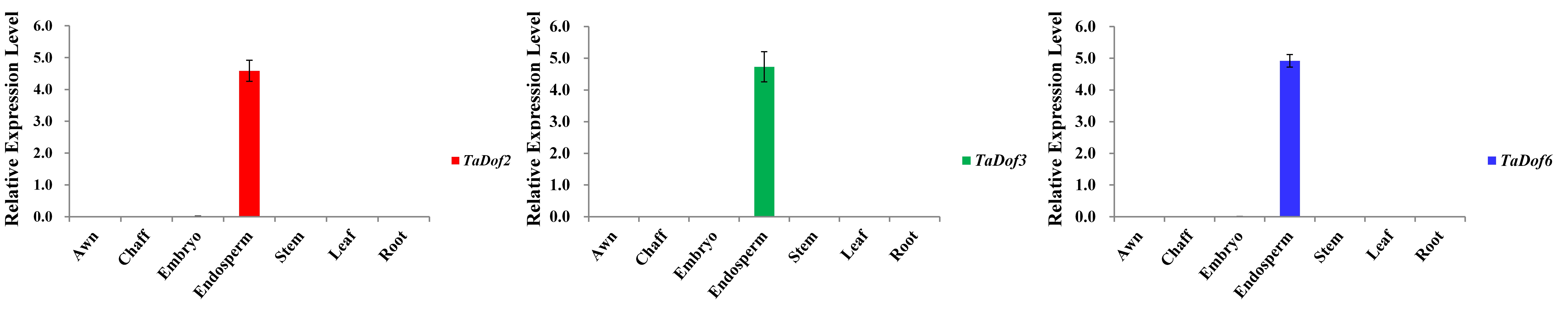

Supplement: Supplementary file 4 — Additional file 4: Figure S4. Expression profiling of TaDof2, TaDof3 and TaDof6 genes in seven wheat tissues and organs. [file 12864_2020_6691_MOESM4_ESM.jpg]
